# Prognostic value of ubiquitin carboxy-terminal hydrolase L1 in patients with moderate or severe traumatic brain injury: a systematic review

**DOI:** 10.1186/cc14534

**Published:** 2015-03-16

**Authors:** M Shemilt, JF Laforest, F Lauzier, A Boutin, D Fergusson, R Zarychanski, L Moore, L McIntyre, L Nadeau

**Affiliations:** 1 CHU de Québec, QC, Canada; 2 Ottawa Health Research Institute, Ottawa, ON, Canada; 3 University of Manitoba, Winnipeg, MB, Canada

## Introduction

Traumatic brain injury (TBI) prognostication is a developing field striving to identify indicators, notably neurochemical biomarkers, of long-term outcomes. Among these, ubiquitin carboxyterminal hydrolase L1 (UCH-L1) is currently being investigated to define its potential prognostic value. The objective of this systematic review is to determine the ability of UCH-L1 to predict prognosis following a moderate or severe TBI.

## Methods

The MEDLINE, Embase, The Cochrane Library and BIOSIS electronic databases, conference abstracts and existing narrative reviews were searched from their inception to July 2013. Cohort studies including patients with moderate or severe TBI having evaluated the prognostic value of UCHL-1 according to mortality or the Glasgow Outcome Scale (GOS) were considered. Data concerning patients, outcomes, study methods, and laboratory methods were abstracted. Pooled results were planned to be presented using mean differences and analyzed using random effect models, as well as sensitivity analyses to explain potential heterogeneity.

## Results

Our search strategy yielded 2,257 articles, of which five studies corresponded to our inclusion criteria (*n *= 730). All studies were performed by the same group of researchers. Five studies reported mortality (*n *= 515), two studies reported GOS (*n *= 58). Results from all included studies observed that UCHL-1 was a significant predictor of mortality. However, only two studies represented a unique study population, thus precluding a meta-analysis.

## Conclusion

In this systematic review, we observed that all published studies on UCHL-1 were conducted by the same group of investigators and presented results from an intersecting cohort of patients. Due to the paucity of data, we could not perform a pooled analysis and conclude on the association of this biomarker with long-term prognosis. Assays using UCHL-1 were only recently developed and further studies done by different research teams will be needed to determine the reproducibility and validity of UCH-L1 as a potential prognostic tool.

